# Unlocking Clinical Precision Through Polygenic Risk Prediction

**DOI:** 10.3126/nje.v14i3.82370

**Published:** 2025-07-27

**Authors:** Brijesh Sathian, Javed Iqbal, Indrajit Banerjee, Hanadi Al Hamad

**Affiliations:** 1 Geriatrics and long term care department, Rumailah Hospital, Hamad Medical Corporation, Doha, Qatar; 2 Nursing Department Hamad Medical Corporation Doha Qatar; 3 Sir Seewoosagur Ramgoolam Medical College, Belle Rive, Mauritius

## Background

Genomic medicine has undergone significant changes over the last decade. The Polygenic Risk Score (PRS), which combines the impact of hundreds to millions of genetic variations to approximate an individual's risk of developing a complex disease (such as coronary artery disease, diabetes, or Alzheimer's disease), is one of the most promising tools. In comparison to monogenic variants, which can carry high and uncommon risks, polygenic models offer predictive capabilities for the population. However, these findings have not yet been translated into clinical care.

Recent achievements have promoted the potential usefulness of PRS. Khera et al. showed that individuals with the highest polygenic score for coronary artery disease were at the same risk level as those with familial hypercholesterolemia [1]. Analogous predictive abilities have been observed in breast cancer, prostate cancer, and atrial fibrillation [2,3]. These results suggest the potential for early screening, pharmacotherapy prophylaxis, and behavioral interventions to occur long before the manifestation of symptoms.

Despite their potential, PRS integration into clinical practice faces significant obstacles. The first limitation is generalizability. Genome-wide association studies (GWAS) employed in the derivation of PRS have primarily been conducted in individuals of European descent. Therefore, PRSs are only applicable to European populations and, when utilized, can exacerbate existing healthcare disparities [4]. When a PRS built on European populations was used to predict individuals of African ancestry, the accuracy of the prediction decreased by more than 80% in one study [5].

Two other major obstacles are interpretability and clinical utility. PRS does not provide definitive information but probabilistic information. In contrast to BRCA mutations, which have straightforward implications for clinical decision-making, the effects of being in a high polygenic risk decile remain complex and uncertain in most cases. Additionally, there is an absolute risk that is dependent on baseline risk factors, including age, sex, and lifestyle; this underscores the necessity of incorporating the PRS into multifactorial risk models, but not interpreting them in isolation.

The PRS ethical world is also complicated. The ethics of informed consent, genetic discrimination, and risks of misuse in the insurance or employment fields have been back on the table with a vengeance. With increasing access to PRS in direct-to-consumer processes, questions regarding proper counseling, data privacy, and interpretation arise. Such a landscape demands a practical regulatory framework to protect people and regulate the responsible use of genetic risk information flow.

The last frontier is the application of PRS in low- and middle-income countries (LMICs), including Nepal, which already experiences overloaded public health systems. Although PRS has the potential to increase accuracy in population-based screening or risk stratification, genomic infrastructure, ethical governance of these tests, and culturally adequate counseling would need to be heavily invested in. In the absence of these precautionary measures, the implementation of PRS can exacerbate health inequality instead of narrowing it [6].

In the future, the integration of PRS with EHRs and AI-based clinical decision support systems could provide a scalable implementation mechanism. This would allow for the possibility of dynamic risk updating as new data are generated and provide clinicians with a contextual interpretation. However, this should be accompanied by ancestry-wide validation, clinical actionability and equity.

In summary, polygenic risk scores represent a promising step in predictive genomics. However, to move PRS beyond research tools to clinically useful measures, the challenges of equity, interpretability, and utility need to be addressed in genomics. It is high time that policymakers and clinicians take action to ensure that PRS are not only empirically sound but also ethical and inclusive worldwide.
